# Diagnostic accuracy of ultrasound characteristics in the identification of malignant thyroid nodules

**DOI:** 10.1186/s13104-019-4235-y

**Published:** 2019-04-02

**Authors:** Mihiri Chami Wettasinghe, Shanthini Rosairo, Neelakanthi Ratnatunga, Nuwan Darshana Wickramasinghe

**Affiliations:** 1 Department of Radiology, Teaching Hospital, Peradeniya, 20400 Sri Lanka; 2 Department of Radiology, Faculty of Medicine, University of Peradeniya, Peradeniya, 20400 Sri Lanka; 3 Department of Pathology, Faculty of Medicine, University of Peradeniya, Peradeniya, 20400 Sri Lanka; 4 Department of Community Medicine, Faculty of Medicine and Allied Sciences, Rajarata University of Sri Lanka, Saliyapura, 50008 Sri Lanka

**Keywords:** Diagnostic accuracy, Ultrasound characteristics, Thyroid malignancy, Microcalcifications, Sri Lanka

## Abstract

**Objective:**

This study was aimed at determining the diagnostic accuracy of the ultrasound characteristics in the identification of malignant thyroid nodules in patients attending the surgical clinic at a tertiary care hospital in Sri Lanka.

**Results:**

This prospective validation study was conducted among 263 patients attending the surgical clinic at a tertiary care hospital, Sri Lanka. The ultrasound characteristics having statistically significant associations with thyroid malignancies were identified by employing a multivariable analysis. These ultrasound characteristics were compared with Fine Needle Aspiration Cytology results and indicators of diagnostic accuracy were computed. The study sample consisted of 33 malignant and 230 benign nodules. Internal vascularity, hypoechogenicity and microcalcification showed statistically significant positive associations with thyroid malignancy (p < 0.05). The highest positive likelihood ratio was observed for the presence of microcalcifications (10.4, 95% CI 4.6–23.7) with a specificity of 96.5% (95% CI 93.3–98.5%). Hypoechogenicity had a sensitivity of 66.7% (95% CI 48.2–82.0%) and a specificity of 74.8% (95% CI 68.6–80.3%). The presence of at least one positive ultrasound characteristic yielded the highest sensitivity (87.9%, 95% CI 71.8–96.6%), whereas, the presence of all three characteristics yielded a near perfect specificity (99.6%, 95% CI 97.6–99.9%). Hence, the presence of microcalcifications was found to be the single most useful criterion in predicting thyroid malignancy.

## Introduction

Globally, thyroid malignancy is the most common endocrine cancer and there has been a steady increase in the global incidence of thyroid cancer over the last few decades [1, 2]. Since there is a 5–15% probability of malignancy in any given thyroid nodule [3, 4], current thyroid guideline recommendations call for imaging modalities to detection of thyroid malignancies early leading to a better prognosis [5]. Ultrasound scanning is widely cited as the imaging modality of choice for early diagnosis owing to its wide availability, relative simplicity and non-exposure to ionizing radiation [6–10].

Despite the highlighted advantages in ultrasound imaging, the evidence is not conclusive in predicting thyroid malignancies [11, 12]. Hence, the diagnostic accuracy of the ultrasound scanning needs to be evaluated by comparing with the results of a gold standard test. Fine needle aspiration cytology (FNAC) is considered as the most reliable cost effective method for definitive evaluation of thyroid nodules [13–20]. Thus, thyroid cancer management guidelines recommend conducting FNAC on any thyroid nodule, which is suspected to be malignant [5, 8–10]. Global evidence highlights a multitude of ultrasound characteristics that are indicative of thyroid malignancies [3, 7–9, 13, 14, 16, 19, 21–30]. However, collective global evidence base suggests inconsistent results across studies on predictive abilities.

Thyroid nodules are common clinical presentations to the surgical clinics in Sri Lanka [31]; hence, accurate identification of potential malignancy will have a great impact on patient management. In this background, it is important to utilise the ultrasound imaging techniques optimally, especially to decide on cases necessitating urgent surgical interventions. Despite the significance, only a limited number of studies have been conducted in Sri Lanka to evaluate the diagnostic accuracy of the thyroid ultrasound imaging [32]. In this research vacuum, the present study was conducted to determine the diagnostic accuracy of the ultrasound characteristics in identification of malignant thyroid nodules.

## Main text

### Methods

The findings are reported following the Standards for Reporting of Diagnostic Accuracy Studies (STARDS 2015) guidelines [33].

#### Study design and setting

This prospective validation study was carried out at the Radiology Department in one of the largest tertiary care centres in the Central Province, Sri Lanka for a period of 18 months from January 2014 to June 2015.

#### Participants

In this single-gate study, all patients referred to the Radiology Department during the study period for thyroid ultrasound scanning were considered as the study population, excluding patients with known bleeding diathesis. All patients who fulfilled the eligibility criteria were recruited for the study upon the informed written consent.

#### Measures

##### Ultrasound scan of the thyroid

Ultrasound scanning was done by an experienced Consultant Radiologist, who has over 15 years experience in thyroid ultrasound scanning. Following criteria for the determination of ultrasound characteristics suggestive of the thyroid malignancies were developed based on the findings of an extensive literature review. The Thyroid Imaging Reporting and Data System (TI-RADS) Committee of the American College of Radiology has presented a risk-stratification system for classifying thyroid nodules on the basis of their appearance at ultrasonography [34] and the five ultrasound characteristics included in this classification were also included in this study.

1. Predominantly solid nodular consistency [21, 25, 34].
2. Hypoechogenicity [8, 9, 14, 16, 19, 21, 24, 34].
3. Absence of a regular border [7, 14, 16, 19, 21, 24, 29, 34].
4. Absence of a halo or presence of an interrupted halo [7, 13, 16, 19, 27].
5. Internal vascularity [7, 13, 16, 19, 28–30].
6. Nodules taller than wide [3, 7–9, 14, 19, 21, 29, 34].
7. Nodule size equal or more than 2 cm [19, 22].
8. Presence of microcalcifications [7–9, 14, 16, 19, 21, 29, 34].

All 263 participants have undergone the ultrasound scanning of the thyroid nodules and the above-mentioned ultrasound characteristics were recorded in each participant.

#### Ultrasound guided FNAC of thyroid nodules

Following the ultrasound scanning, participants were subjected to ultrasound guided FNAC, upon their informed written consent. The FNAC of the thyroid nodules were carried out by the same Consultant Radiologist, who performed the ultrasound scanning, using the same ultrasound scanner used to detect the nodule. The procedure was done under strict aseptic conditions using a 23G needle and three samples were taken from each nodule. Prepared slides were immersed immediately in 95% ethyl alcohol solution, before transporting them to the laboratory.

#### Cytology of thyroid nodules

The thyroid cytopathology report was prepared according to the Bethesda classification [35]. An experienced Consultant Pathologist (with more than 25 years experience in histopathology), who was blinded to the ultrasound scan findings, evaluated all the samples.

Data analysis was carried out by using Statistical Package for the Social Sciences (SPSS) analytic software version 25.0. Based on FNAC findings, the sample was categorised as benign and malignant.

To identify the ultrasound characteristics with significant associations with thyroid malignancy, a bivariate analysis was conducted and the crude odds ratios (OR) were computed with 95% confidence intervals (95% CI). A binomial multiple logistic regression model was used to control for confounding. Ultrasound characteristics that showed statistical significance (p < 0.05) in the bivariate analysis were included in the multivariable analysis model using backward stepwise elimination method and the model produced adjusted odds ratios (AOR) and 95% CI.

The statistically significant ultrasound characteristics were compared with FNAC results separately and in combination. Performance characteristics of each ultrasound characteristic and combined ultrasound characteristics, namely, sensitivity, specificity, positive and negative predictive values and the positive likelihood ratios (LR+) were computed. Point estimates for the performance characteristics were supported by the 95% CI estimates.

### Results

#### Characteristics of the sample

The study sample consisted of 263 participants and all participants have undergone both ultrasound scanning and ultrasound guided FNAC. The majority of participants were females (n = 247, 93.9%). The age range of the sample was 16 years to 74 years (mean = 46.1 years, SD = 12.7 years).

#### Diagnosis of the ultrasound guided FNAC

Based on the cytological diagnosis of the thyroid nodules, the sample was categorised as benign and malignant. According to the FNAC results, Thy2 lesions were categorised as benign (n = 163). In addition, the benign category consisted of the Thy3 and Thy4 lesions (n = 79), which underwent surgery (n = 58) and histopathologically confirmed as benign (n = 46). Further, Thy3 lesions, which showed no interval changes after 6 months, were also included in this category (n = 21). The malignant group consisted of all the histological proven malignancies in Thy3 and Thy4 group (n = 12) and those that were Thy5 (n = 10) and Thy6 (n = 11) from the FNAC results. Thus, there were 33 proven malignant thyroid nodules and 230 benign nodules.

#### Association between the ultrasound characteristics and thyroid malignancy

Table 1 summarises the results of the bivariate analysis of the association between ultrasound characteristics and thyroid malignancy and out of the eight ultrasound characteristics assessed, except for the size and consistency, other characteristics showed statistically significant associations with thyroid malignancy at p < 0.05.

**Table 1:** Association between ultrasound characteristics and thyroid malignancy in the bivariate analysis (n = 263)

Ultrasound characteristic	Malignant, n (%)	Benign, n (%)	Total, n (%)	Crude odds ratio (95% CI)	p value
Size (cm)					
≥ 2	14 (12.1)	102 (87.9)	116 (100.0)	0.9 (0.4–1.9)	0.854
< 2	19 (12.9)	128 (87.1)	147 (100.0)		
Taller than wide					
Taller than wide	16 (18.8)	69 (81.2)	85 (100.0)	2.3 (1.0–4.6)	0.046
Wider than tall	17 (9.6)	161 (90.4)	178 (100.0)		
Echogenicity					
Hypoechogenicity	22 (27.5)	58 (72.5)	80 (100.0)	5.9 (2.7–12.9)	< 0.001
Iso/hyperechogenicity	11 (6.0)	172 (94.0)	183 (100.0)		
Consistency					
Solid	32 (13.7)	202 (86.3)	234 (100.0)	4.4 (0.6–33.7)	0.145
Cystic	1 (3.4)	28 (96.6)	29 (100.0)		
Borders					
Irregular	12 (38.7)	19 (61.3)	31 (100.0)	6.3 (2.7–14.9)	< 0.001
Regular	21 (9.1)	211 (90.9)	232 (100.0)		
Halo					
Absent halo	26 (17.9)	119 (82.1)	145 (100.0)	3.5 (1.4–8.3)	0.004
Halo present	7 (5.9)	111 (94.1)	118 (100.0)		
Calcification					
Micro-calcification	12 (60.0)	8 (40.0)	20 (100.0)	15.9 (5.8–43.1)	< 0.001
No micro-calcification	21 (8.6)	222 (91.4)	243 (100.0)		
Vascularity					
Internal vascularity	16 (25.0)	48 (75.0)	64 (100.0)	3.6 (1.7–7.6)	0.002
No internal vascularity	17 (8.5)	182 (91.5)	199 (100.0)		

Table 2 illustrates the results of the analysis of binary logistic regression of the ultrasound characteristics retained in the final model. Out of the five factors retained in the final model, three factors made unique statistically significant contributions at p < 0.05. When controlled for other factors included in the logistic regression analysis, internal vascularity, hypoechogenecity and microcalcification showed statistically significant positive associations with thyroid malignancy.

**Table 2:** Results of the logistic regression analysis to elicit the association of ultrasound characteristics and thyroid malignancy (n = 263)

Ultrasound characteristic	B	SE	Wald	*df*	p value	Adjusted OR	95% CI
Taller than wide	0.912	0.473	3.710	1	0.054	2.5	1.0–6.3
Hypoechogenicity	1.533	0.469	10.679	1	0.001	4.6	1.8–11.6
Absent halo	0.914	0.534	2.924	1	0.087	2.5	0.9–7.1
Microcalcification	3.037	0.640	22.545	1	< 0.001	20.8	5.9–72.9
Internal vascularity	1.090	0.458	5.662	1	0.017	2.9	1.2–7.3

*95% CI* 95% confidence interval, *df* degree of freedom, *OR* odds ratio, *SE* standard error

#### Diagnostic accuracy of ultrasound characteristics in the identification of thyroid malignancies

The performance characteristics of individual ultrasound characteristic evaluated against the final diagnosis are summarised in the Table 3.

**Table 3:** Summary of indicators of diagnostic accuracy of ultrasound characteristics in the identification of thyroid malignancy

Ultrasound characteristic	Sensitivity (95% CI)	Specificity (95% CI)	PPV (95% CI)	NPV (95% CI)	LR+ (95% CI)
Hypoechogenicity	66.7% (48.2–82.0%)	74.8% (68.6–80.3)	27.5% (18.1–38.6%)	94.0% (89.5–97.0%)	2.6 (1.9–3.7)
Microcalcification	36.4% (20.4–54.9%)	96.5% (93.3–98.5%)	60.0% (36.0–80.9%)	91.4% (87.1–94.6%)	10.4 (4.6–23.7)
Internal vascularity	48.5% (30.8–66.5%)	79.1% (73.3–84.2%)	25.0% (15.0–37.4%)	91.5% (86.7–94.9%)	2.3 (1.5–3.6)
At least one positive characteristic	87.9% (71.8–96.6%)	58.7% (52.0–65.1%)	23.4% (20.0–27.1%)	97.1% (93.1–98.8%)	2.1 (1.7–2.6)
At least two positive characteristics	54.5% (36.4–71.9%)	92.2% (87.9–95.3%)	50.0% (36.8–63.2%)	93.4% (90.7–95.4%)	7.0 (4.1–12.0)
All three positive characteristics	9.1% (1.9–24.3%)	99.6% (97.6–99.9%)	75.0% (24.3–96.6%)	88.4% (87.3–89.5%)	20.9 (2.2–195.2)

*95% CI* 95% confidence interval, *PPV* positive predictive value, *NPV* negative predictive value, *LR+* positive likelihood ratio

Amongst the single ultrasound characteristics assessed, the highest LR+ was observed for the presence of microcalcifications and it had a very high specificity, but the sensitivity was relatively low. On the contrary, hypoechogenicity had a relatively higher sensitivity and specificity values.

Amongst the combined ultrasound characteristics, the presence of at least one positive characteristic yielded the highest sensitivity, whereas, the sensitivity values were substantially lower when more than one characteristic was combined. On the contrary, presence of all three characteristics yielded a near perfect specificity.

### Discussion

Since the existing literature on diagnostic accuracy of ultrasound characteristics in predicting thyroid malignancies fails to provide conclusive evidence, we considered a number of important ultrasound characteristics cited in the literature for the present study.

Since experienced consultants in the relevant fields interpreted the findings of the index test and the reference standard separately based on clear, explicit guidelines, the risk of misclassification bias is minimised in the study. Furthermore, given that a single consultant carried out interpretation of ultrasound imaging and cytology separately, there was no inter-observer bias in gathering information. Since the Consultant Pathologist was blinded to the results of the ultrasound scan findings of the participants, there was no risk of bias in interpreting the reference standard test.

In the present study, ultrasound scanning was considered as the index test and FNAC diagnosis, which is cited as the gold standard diagnosis in thyroid malignancies [14, 15, 17–20] was used as the reference standard.

Rather than evaluating all ultrasound characteristics for their diagnostic accuracy, statistically significant characteristics were identified by employing both bivariate analysis followed by the multivariable analysis. This analysis was important in addressing the potential confounding effect of ultrasound characteristics on each other and has aided in determining the most significant characteristics to be used in diagnostic accuracy evaluation. The analysis has yielded three significant ultrasound characteristics, namely, microcalcifications, hypoechogenicity and internal vascularity. These findings are consistent with most of the existing literature, as many studies have revealed satisfactory indicators of diagnostic accuracy of microcalcification [7–9, 14, 16, 19, 21, 29], hypoechogenicity [8, 9, 14, 16, 19, 21, 24] and internal vascularity [7, 13, 16, 19, 28–30] in predicting thyroid malignancy.

The presence of microcalcifications is associated with a LR+ of 10.4 (95% CI 4.6–23.7), indicating that the likelihood of having thyroid malignancy has increased by approximately ten-fold given the positive ultrasound characteristic. However, this characteristic was found to have a low sensitivity of 36.4%, which is consistent with low sensitivity (26.1–59.1%) values reported in the literature [3, 14, 16, 21, 29]. In contrast, the specificity of this ultrasound characteristic was very high (96.5%), which is consistent with previously reported values of 90.8% [21] and 87.8% [19]. The strong association of microcalcifications in thyroid malignancies is thought to be due to the fact that microcalcifications correspond pathologically to calcified ‘psammoma bodies’ that are typical of papillary cancer [21, 36, 37]. In contrast, macrocalcifications or coarse calcifications are related to fibrosis and degeneration [38, 39] and benign nodules of long duration are mostly associated with coarse calcifications [40, 41].

In the present study, hypoechogenicity was shown to have a sensitivity of 66.7% and a specificity of 74.8%. These findings are congruent with existing literature, in which the sensitivity was reported as 62.7% in a meta-analysis [19]. However, literature suggests that the sensitivity values range from 26.5% [14] to 87.2% [21]. In a similar vein, even though the meta-analysis findings reveal a specificity of 62.3% [19], the values range from 58.5% [21] to as high as 94.3% [14]. Furthermore, the LR+ reported in the present study is similar to that in the meta-analysis [19].

The study findings reveals that internal vascularity had a sensitivity of 48.5%, specificity of 79.1% and a LR+ of 2.3, corresponding with the meta-analysis findings of 45.9%, 78.0% and 2.1 of sensitivity, specificity and LR+ respectively [19]. On the contrary, some studies have concluded that the intra-nodular colour flow alone has a lower predictive value in assessing the malignant thyroid nodules [13, 42].

In relation to the combined ultrasound characteristics, the presence of at least one positive characteristic yielded the highest sensitivity, whereas, the presence of all three characteristics yielded a near perfect specificity. Furthermore, when considering multiple ultrasound characteristics in combination, the LR+ of detecting malignant thyroid nodules increased. These findings are congruent with the findings of the existing literature [16, 32].

Our study showed that the presence of microcalcifications and hypoechogenicity are the most significant criteria in predicting thyroid malignancies, which are also included in the TI-RADS classification. In addition to the characteristics included in the TI-RADS classification, internal vascularity has shown a statistically significant association in our study. Thus, further studies are needed to explore the role of vascularity in predicting thyroid malignancies.

## Limitations

In this study, all the subjects with benign FNAC results did not attend the follow-up ultrasound scan in 6 months. Of Thy3 and Thy4 lesions, 58 subjects underwent surgical removal and the final diagnosis was obtained from the histopathological report. Twenty-one subjects with Thy3 lesion did not undergo surgery, but attended follow-up ultrasound scan.

## Abbreviations

AOR: adjusted odds ratio
FNAC: fine needle aspiration cytology
LR+: positive likelihood ratio
OR: odds ratio
SD: standard deviation
TI-RADS: thyroid imaging reporting and data system
95% CI: 95% confidence interval
